# Effect of highly active antiretroviral treatment on TB incidence among HIV infected children and their clinical profile, retrospective cohort study, South West Ethiopia

**DOI:** 10.1038/s41598-020-78466-0

**Published:** 2020-12-08

**Authors:** Firew Tiruneh, Yared Deyas

**Affiliations:** Department of Midwifery, College of Health Science, Mizan Tepi University, PO box 260, Mizan Teferi, Ethiopia

**Keywords:** Immunology, Microbiology

## Abstract

Globally, 1.8 million HIV infected children live with HIV; nearly 53% of them were receiving HIV treatment. People who are infected with HIV are 18 times more likely to develop active TB. Despite antiretroviral treatment has shown marked reduction in TB incidence, TB continues to occur in Sub-Saharan countries including Ethiopia among HIV infected people. The effect of highly active antiretroviral treatment is quite successful in developed countries. However, in developing country TB/HIV co-infection remains perplexing among children on the treatment. The aim of this study was to investigate the impact of ART on the incidence of TB among Children infected with HIV in Southwest Ethiopia. A retrospective cohort study was conducted on randomly selected 800 samples from ART clinic between 2009 and 2014. We used chi-square test, and Mann–Whitney U test to compare HAART naïve and HAART cohort. We used marginal structural models to estimate the effect of HAART on survival while accounting for time-dependent confounders affected by exposure. A total of 800 children were followed for 2942.99 child-years. The children were observed for a median of 51 months with IQR 31 and for a total of 2942.99 child-years. From 506 OIs that occurred, the most common reported OIs were Pneumonia (22%) and TB (23.6%). The overall TB incidence rate was 7.917 per 100 child years (95% CI, 6.933–9.002). Whereas among HAART (7.667 per 100-years (95% CI, 6.318–9.217) and 8.1686 per 100 person-years (95% CI 6.772–9.767) for HAART naïve. The mortality hazard ratio comparing HAART with no HAART from a marginal structural model was 0.642 (95% CI 0.442–0.931, *p* < 0.02). HAART reduced the hazard of TB in HIV-infected children by 36%. This is by far less than expected.

## Introduction

Globally, around 38 million peoples were expected to live with HIV at the end of 2019^1^. In the same year 1.8 million children estimated to live with HIV. Of 1.8 million only 950 000 (53%) of them were receiving HIV treatment, much lower than the 67% of adults on treatment^2^. People who are infected with HIV are 18 times more likely to develop active TB. Worldwide TB is the most common opportunistic illness and the leading cause of death among HIV infected people^3,4^. In 2019, 1.2 million children fell ill with TB globally^5^. According to WHO, there were an estimated 0.9 million new cases of TB amongst people who were HIV-positive, 72% of whom were living in Africa^4^. studies have estimated 15–20% of TB cases among children in settings with a high burden of TB^6,7^.

HIV and TB form a lethal combination, each speeding the other's progress^8,9^. People with Latent TB have lifetime risk of 10–15% to develop active TB disease. The risk of developing TB in HIV-infected children is 20 times higher than HIV-uninfected children^10^. TB risk is higher among HIV infected children. Without treatment, 15–50% of HIV-positive infants and children will develop active TB within two years after becoming infected with TB^11^.

To prevent TB, HAART is one of the strategies recommended by the World Health Organization (WHO).TB^12,13^. HAART has shown marked reduction in the incidence of TB(1)^14–16^. Although the risk of developing TB is reduced by 70–90% among HIV-infected persons receiving HAART, compared with untreated individuals^17^. However, TB risk was reported to be high following HAART initiation^18,19^.

Higher risk may be partially due to incomplete immune restoration and “unmasking” of previously undiagnosed TB in the setting of immune reconstitution^20^. The effect of HAART on TB incidence in resource constrained country may be adversely affected by multiple factors including delayed presentation to care and a higher incidence of co-occurring infectious and non-infectious conditions such as under nutrition and adherence level^14,20^.

HIV-infected children are most at risk to develop TB. Globally, from estimated 10.0 million new TB infection, 1 million of them were children less than 15 years age, in 2017^21^. In Ethiopia, reported number of PLHIV treated with HAART were 71% and out of this 32% have suppressed viral load measurement in 2017^22^. TB case notification for TB was 6% with rapid diagnostic tool while TB preventive treatment reached for 47%^23^. Routine screening and treatment for latent TB infection (LTBI) has been shown to reduce the risk of active TB in HIV-infected patients^24^.

As to our understanding, studies on the possible effect of HAART on HIV-associated TB remains limited. We are aware that there are few studies in Ethiopia that tried to see the incidence of TB in the same population^7,25,26^. However, their analysis was flawed. We estimated effect of HAART on the incidence of TB among HIV infected children using marginal structural regression modeling after adjusting and weighting baseline and time varying covariates.

## Methods

### Study design and setting

A retrospective cohort study was conducted. We reviewed patient chart, clinical record and ART data base for five years from 2009 to 2014. The study was conducted in selected ART clinics found in southwest Ethiopia. These ART clinics were randomly selected using simple random sampling. South West Ethiopia encompasses five zones namely, Jimma, Illu Ababora, Kafa, Sheka and Bench-Maji.

### Definitions

- In our study we defined TB case as: (1) Smear positive result of acid fast bacilli or (2) TB confirmed by culture or (3) Clinical sign and symptoms compatible with TB infection criteria by WHO or (4) Chest radiography confirmation.
- Children on HAART was defined as those who took combination therapy of 3 antiretroviral drugs that included two non-nucleoside reverse transcriptase and one protease inhibitor.
- Children, who were lost, died, or transferred out or did not develop the events until the last visit were considered as censored.

### Source and study population

All children younger than 15 years having a follow up care in ART clinic in south west Ethiopia were source population. While all randomly selected HIV positive under 15 years old children registered from September 2009 to August 2014 in south west Ethiopia were study population.

- Exposure—treatment with highly active antiretroviral treatment (HAART) for at least 2 month.
- Outcome—TB illness.

### Inclusion and exclusion criteria

- Inclusion—All under 15 years old children on HAART or Pre-HAART follow up who were registered from September 2009 to August 2014.
- Exclusion—All under 15 years old children who started anti-TB treatment at the beginning of follow up and/or diagnosed as TB patient.

### Sample size and sampling procedure

844 sample size was calculated with double population proportion formula by considering the following assumption.

α = Type one error (0.05).

Z α/2 = Critical value at 95% level of significance.

Z1-β = standard normal distribution value corresponding to power (90%).

Ration = 1:1

Fresh list of hospitals in each zone namely Bench Maji, Kefa, Sheka, Jimma and Ilubabor were prepared. After having the list of ART clinics that provide ART service for less than 15 years of age, and with proportional allocation methods, samples were selected from each ART clinic with systematic random sampling technique for both ART and Pre ART cohort.

### Data collection tool and procedure

A standardized tool which has been adapted from existing literature was used. The tool was translated to local language by language expertise. Then relevant data was collected from patients’ pre-ART and ART follow up log books, Data base and other clinical records. Data was collected by six data clerk who work in ART clinic after receiving 2 days training on how to extract data from these records. To assure quality of data extraction, a pretest was conducted in 5% of the sampled population.

### Data entry and analysis

The collected data were coded and double entry was made in EpiData version 3.1 statistical package. Then the data were exported to the Statistical Package for the Social Sciences (SPSS) version 23 software for analysis. Before analysis, the data were processed and cleaned by running frequency, sorting and cross tabulation to check completeness, outliers and data entry errors. Descriptive statistics analysis were done to describe the characteristics of the study subjects.

To compare proportions of TB between HAART naïve and HAART cohort, we used the chi-square test, whereas we compared medians by the Mann–Whitney test. The Incidence rate was calculated with an Open Epi software and were expressed per 100 child-years of follow-up on HAART. The TB free survival probability were constructed by the Kaplan–Meier method and compared between HAART naïve and HAART groups by the log-rank test. The comparisons was two-tailed with *p* values < 0.05.

We estimated the effect of HAART on TB incidence by adjusting for confounders measured at baseline and time-varying intermediates. We did this by fitting weighted pooled logistic regression model to construct stabilized inverse probability of treatment and censoring (IPTC) weights. Then, these IPTC weights were used in a weighted pooled logistic regression model to approximate the parameters of a marginal structural Cox model^27^. Each patient in the above logistic models received a time-varying weight inversely proportional to the estimated probability of having his/her own observed history of HAART initiation, as described elsewhere. For each child and visit, the denominator of the weight can be viewed as the probability that they received their actual treatment history and remained uncensored up to that time, conditional on their past treatment and covariate history. Confounders were selected based on previous studies. The covariates included in these models were age, sex, baseline clinical stage and baseline CD4 as baseline covariates. The time varying covariates were CD4, Clinical stage, follow-up status, adherence and CPT.

Bivariable and multivariable Cox proportional hazard regression models were used to see independent predictors of TB incidence. Variables with *p* value < 0.2 in bivariable analysis were transferred to Multivariable cox proportional hazard model. In Multivariable analysis, Variables with *p* value < 0.05 at 95% confidence level was considered as statistically significant predictors of TB incidence. The results were expressed as hazard ratios (HRs) with 95% confidence intervals (CI).

### Ethics approval and informed consent

Ethical clearance was obtained from the Institutional Review Board (IRB) of Mizan-Tepi University. Permissions was obtained from Bench Maji, Kefa, Sheka, Jimma and Ilubabor Zonal Health Department. Moreover, a Verbal permission was obtained from selected facility administration before starting data collection. Informed Written consent was obtained from parents or legal guardian during the period of data collection. The study was performed in accordance with the relevant guideline and regulation.

## Results

From the total estimated sample size (844) around 94.8% children were retrospectively followed. Half of them, 50% were HAART naïve while 50% were HAART initiated. The median follow up period for HAART naïve was 47 months with IQR of 31 months whereas HAART initiated were followed for a median of 53 months with IQR of 32 months. The total follow up period for HAART naïve children and HAART initiated were 1469.08 and 1473.91 child year time respectively. The median and inter-quintile range (IQR) of age for HAART cohort were 9 and 6 years respectively. The corresponding values for the Pre-ART cohort were 7 and 5 years respectively. In terms of gender males constitute the largest proportion in both HAART (51%) and HAART naïve (56.8%) groups.

During a total of 2942.99 person-years follow-up, 506 OIs occurred in 248 children (31%). The most common reported OIs were Pneumonia (22%) and TB (29.1%). Nearly 2% children died, 7.8% transferred out, 12.3% lost to follow up. Of 12.3% lost to follow up 35.7% were female, and the rest 64.3% were male. Sixty five percent of the study subjects were either in WHO clinical stage I or II, while 65.3% had a CD4 count above threshold at enrollment.

Baseline characteristics, children on HAART compared to those HAART naïve later had a degree of advanced WHO clinical stage (*p* < 0.001). Children who had a baseline cd4count below threshold, had a more advanced HIV clinical stage (*p* = 0.03), and were more likely to have TB (*p* < 0.01). Although those who initiated HAART had no difference as compared to those who did not regarding gender (*p* = 0.10), there was a difference regarding the medians of age across categories of HAART treatment (*p* < 0.001). The characteristics of study subjects are indicated in Table 1.

**Table 1:** Characterstics of HAART initiated and HAART naïve children.

Time period	Characteristics	Category	HAART naïve		HAART initiated	
			TB (no)	TB (yes)	TB (no)	TB (yes)
Baseline variables	Age	< 1	3 (27.3%)	8 (72.7%)	0 (0%)	18 (100%)
		1–3	56 (59.6%)	38 (40.4%)	21 (48.8%)	22 (51.2%)
		3–5	45 (81.8%)	10 (18.2%)	41 (67.2%)	20 (32.8%)
		5–15	176 (73.3%)	64 (26.7%)	225 (80.9%)	53 (19.1%)
	Sex	Male	148 (65.2%)	79 (34.8%)	127 (62.3%	77 (37.7%)
		Female	132 (76.3%)	41 (23.7%)	160 (81.6%)	36 (18.4%)
	WHO clinical stage	I	158 (75.2%)	52 (24.8%)	152 (78.8%)	41 (21.2%)
		II	106 (74.1%)	37 (25.9%)	95 (70.9%)	39 (29.1%)
		III	16 (34.0%)	31 (66.0%)	34 (57.6%)	25 (42.4%)
		IV	0 (0%)	0 (0%)	6 (42.9%)	8 (57.1%)
	CD4	Below threshold	73 (62.9%)	43 (37.1%)	103 (65.2%)	55 (34.8%)
		Above threshold	207 (72.9%)	77 (27.1%)	184 (76.0%)	58 (24.0%)

*WHO* world health organization, *TB* Tuberculosis.

During the 2942.99 person-years of follow-up, 189 children developed TB .The overall TB incidence rate was 7.917 per 100 child years (95% CI, 6.933–9.002). Whereas among HAART 7.667 per 100-years (95% CI, 6.318–9.217) and 8.1686 per 100 person-years (95% CI 6.772–9.767) for HAART naïve. The medians of time to event observation for children who started HAART was statistically similar with the medians observed for untreated children (*p* = 0.229). In comparison the proportion of HAART naïve children who developed TB (29.5%) were higher than the 17.8% of HAART-initiated. However, no significant statistical difference was observed in terms of TB incidence between children on HAART and HAART naïve (*p* = 0.641).

The risk of TB was higher among children whose CD4 cell count was below threshold as compared to those the corresponding value was above threshold (*p* = 0.003). However, there was similar distribution of CD4 count between children who developed TB and who does not (*p* = 0.744). The incidence of TB has a direct relationship with age, as the age increase the risk also increases (*p* < 0.001).

An unadjusted model that did not account for any confounder estimated no protective effect of HAART on TB incidence, relative to HAART naïve (HR 1.019, 95% CI 0.788–1.318, *p* = 0.885). The unweighted model that adjusted baseline confounder suggested (HR 0.933, 95% CI 0.98–0.712–1.224, *p* = 0.618). Similarly when we fit weighted model appropriately accounting for base line confounder the result does not suggest protective effect of HAART (HR, 0.903, 95% CI 0.657–1.240, *p* = 0.529). However, the model that accounted both the baseline and time-varying confounders using marginal structural models showed that HAART reduces the risk of TB; the HR from IPTC-weighted model was (HR, 0.642, 95% CI 0.442–0.931, *p* = 0.020) (Table 2).

**Table 2:** Estimated effect of HAART on TB incidence among 800 HIV-infected children initiating HAART, southwest Ethiopia, 2010–2014.

Model	HR	95% CI	*p* Value
Unweighted and unadjusted	1.019	0.788–1.318	0.885
Unweighted but adjusted	0.933	0.712–1.224	0.618
Baseline weighted and stabilized	0.903	0.657–1.240	0.529
Time-varying weighted and stabilized	0.683	0.490–0.952	0.024
Total weighted and stabilized	0.642	0.442–0.931	0.020

## Discussion

This study estimated the effect of HAART on TB incidence in HIV infected children on HAART. The finding showed that HAART noticeably had a protective effect against TB among HIV infected children in Southwest Ethiopia. Using a marginal structural model, we estimated that HAART reduced the hazard (rate) of TB incidence by 36% relative to HAART naïve. Although our methods of analysis was different, this finding was supported by a study that estimated TB incidence among children after HAART initiated^28–30^. Our finding was in line with the effect observed with similar methods of analysis, between adults in Europe and the United States^31^. The similarity of these findings offers evidence that HAART is effective against TB in HIV-infected children in SWE. This agreement is important because virtually all children are believed to receive HAART around the world, because of scale-up of antiretroviral provision.

The effect of HAART estimated in our study was lower than the effect revealed by similar studies in in high income countries^32,33^. This different could be resulted from the fact that, Pediatric HIV programs in Southwest Ethiopia like sub-Saharan Africa, often face challenges that could adversely increase TB among HIV infected children, including limited diagnostic capacity, delayed healthcare seeking and poor retention in care and under-nutrition^34^. These factors underline why a similar protective effect of HAART on TB in children could not be expected across different settings.

The incidence of TB among children on HAART in this study was 3.59 per 100 child-years and for HAART naïve group was 4.63 per 100 child-years. Mathematically it seems low; however there is no statistically significant difference between the two groups. This finding is different from studies that supported reduced incidence of TB among HAART initiated^15,35,36^^.^

In the present study high TB incidence was observed among CD4+ count strata below threshold for age during follow-up period. The result depicted an opposite relationship between duration on HAART and TB incidence. This finding is consistent with other study, and this may be due to better TB-specific immune repair with time spent on HAART^37^^.^ Furthermore, in the first few months after HAART initiation we indicated highest rates of TB incidence, possibly due to “unmasked” infection, in the first three months after HAART initiation. Besides, the persistent high rate of new TB infections even after 24 months of HAART initiation were clearly indicated in published studies^38^^.^ We demonstrated that the high TB incidence rates observed in this study may be due to high ongoing community level TB transmission. Poor knowledge of the community and poor health care access. Most importantly this might be justified with our selection criteria. We selected cases with all diagnostic criteria including clinical criteria. The criteria might be less specific but highly sensitive.

Our study has some limitations. In our selection criteria we suspected that we imposed unintentional classification of prevalent TB as incident TB. Children who received no screening for TB in their enrolment were followed and if in the meantime TB was developed then it was considered as incident cases. Besides HAART was not randomly assigned, and thus the possibility of residual confounding cannot be ruled out. We also acknowledge that using Clinical TB diagnostic criteria was the limitation of our study. We were forced to use this criteria as a result of scares resource for diagnosis in the study area. This was most common practiced diagnostic criteria to initiate anti TB treatment.

## Conclusions

The effect of HAART on TB incidence among HIV infected children in this study is analyzed using marginal structural modeling. The study revealed the extent to which therapy decreases TB incidence in a highly relevant population of HIV-infected children living in Southwest setting. In this study HAART reduced the hazard of TB in HIV-infected children by 36%. However, this is by far less than what is expected. TB incidence in the study area was relatively high enough to be considered as a public health problem.

### Recommendation

ART program managers and coordinators shall improve and strengthen the program. By designing strategies and tactical approach that address lost to follow up and drop, TB screening and diagnostic capacity and adherence level. We would also like to recommend to researchers to identify factors affecting HAART effectiveness.

## Data Availability

The datasets during and/or analysed during the current study available from the corresponding author on reasonable request.

## References

[1] Organization WH. HIV/AIDS. Geneva. 2020 [cited 2020 Oct 16]. Available from: https://www.who.int/health-topics/hiv-aids/#tab=tab_1

[2] Children | UNAIDS. Despite great progress since the early days, the HIV response is still failing children [Internet]. 2020 [cited 2020 Oct 16]. Available from: https://www.unaids.org/en/keywords/children

[3] Antiretroviral Therapy Cohort Collaboration (2018). Incidence of tuberculosis among HIV-infected patients receiving highly active antiretroviral therapy in Europe and North America. Clin. Infect. Dis..

[4] World health organization. GLOBAL TB REPORT 2016 WITH HIV + TB. 2016.

[5] World Health Organization. Tuberculosis fact sheet [Internet]. Geneva. 2020 [cited 2020 Oct 16]. Available from: https://www.who.int/news-room/fact-sheets/detail/tuberculosis

[6] Centers for Disease Control and Prevention. TB in specific populations.

[7] Beshir MT, Beyene AH, Tlaye KG, Demelew TM (2019). Incidence and predictors of tuberculosis among HIV- positive children at Adama Referral Hospital and Medical College, Oromia, Ethiopia: a retrospective follow-up study. Epidemiol. Health.

[8] Time to act. Save a million lives by 2015. Prevent and treat tuberculosis among people living with HIV.

[9] Venturini E (2014). Tuberculosis and HIV co-infection in children. BMC Infect. Dis..

[10] Reddi A (2005). Preliminary outcomes of a paediatric highly active antiretroviral therapy cohort from KwaZulu-Natal, South Africa. BMC Pediatr..

[11] Mu W (2014). HIV Incidence and associated factors of pulmonary tuberculosis in HIV-infected children after highly active antiretroviral therapy (HAART) in China: a retrospective study. AIDS Care Psychol. Sociomed. Asp. AIDS.

[12] Lawn SD, Wood R, De Cock KM, Kranzer K, Lewis JJCG (2010). Antiretrovirals and isoniazid preventive therapy in the prevention of HIV-associated tuberculosis in settings with limited health-care resources. Lancet Infect Dis..

[13] STOP TB Partnership. The global plan to stop TB 2006–2015.

[14] Edmonds A (2011). The effect of highly active antiretroviral therapy on the survival of HIV-infected children in a resource-deprived setting: a cohort study. PLoS Med..

[15] Anígilájé EA, Aderibigbe SA, Adeoti AO (2016). Tuberculosis, before and after antiretroviral therapy among HIV-infected children in Nigeria: what are the risk factors?. PLoS ONE.

[16] Santoro-lopes G, Maria A, De PF, Harrison LH (2002). Reduced risk of tuberculosis among brazilian patients with advanced human immunodeficiency virus infection treated with highly active antiretroviral therapy. Clin. Infect. Dis..

[17] Article M (2005). Incidence of tuberculosis among HIV-infected patients receiving highly active antiretroviral therapy in Europe and North America. Clin. Infect. Dis..

[18] Lawn SD, Myer L, Bekker LGWR (2006). Burden of tuberculosis in an antiretroviral treatment programme in sub-Saharan Africa: impact on treatment outcomes and implications for tuberculosis control. AIDS.

[19] Lawn SD, Badri MWR (2005). Tuberculosis among HIV-infected patients receiving HAART: long term incidence and risk factors in a South African cohort. AIDS.

[20] Lawn SD, Wilkinson RJ, Lipman MCWR (2008). Immune reconstitution and “unmasking” of tuberculosis during antiretroviral therapy. Am. J. Respir. Crit. Care Med..

[21] UNICEF. Tuberculosis is now the leading cause of death from infectious diseases for children of all ages globally (2017).

[22] World Health Organization. HIV country profile (2017).

[23] Countries H. Country profiles.

[24] Akolo C, Adetifa I, Shepperd SVJ (2010). Treatment of latent tuberculosis infection in HIV infected persons. Cochrane Database Syst. Rev..

[25] Alemu YM, Awoke W, Wilder-smith A (2016). Determinants for tuberculosis in HIV-infected adults in Northwest Ethiopia: a multicentre case – control study. BMJ Open.

[26] Ayalaw SG, Alene KA, Adane AA (2015). Incidence and predictors of tuberculosis among HIV positive children at University of Gondar Referral Hospital, Northwest Ethiopia: a retrospective follow-up study. Int. Sch. Res. Notices.

[27] Fewell Z (2004). Controlling for time-dependent confounding using marginal structural models. Stata J. Promot. Commun. Stat. Stata.

[28] Palladino C (2009). Impact of highly active antiretroviral therapy (HAART) on AIDS and death in a cohort of vertically HIV type-1 infected children: 1980–2006. AIDS Res. Hum. Retroviruses.

[29] Nesheim SR (2015). Trends in opportunistic infections in the pre- and post-highly active antiretroviral therapy eras among HIV-infected children in the perinatal AIDS. Pediatrics.

[30] Sa M (2003). Impact of highly active antiretroviral therapy on the morbidity and mortality in Spanish human immunodeficiency virus-infected children. Pediatr. Infect. Dis. J..

[31] HIV-CAUSAL Collaboration (2010). The effect of combined antiretroviral therapy on the overall mortality of HIV-infected individuals. AIDS.

[32] Kirk O, Gatell JM, Mocroft A (2000). Infections with *Mycobacterium tuberculosis* and *Mycobacterium avium* among HIV-infected patients after the introduction of highly active antiretroviral therapy. EuroSIDA Study Gr JD. Am. J. Respir. Crit. Care Med..

[33] Badri M, Wilson DWR (2002). Effect of highly active antiretroviral therapy on incidence of tuberculosis in South Africa: a cohort study. Lancet.

[34] De Baets AJ, Ramet J, Msellati PLP (2008). The unique features of pediatric HIV-1 in sub-Saharan Africa. Curr. HIV Res. PLoS.

[35] Walters E (2008). Clinical presentation and outcome of tuberculosis in human immunodeficiency virus infected children on anti-retroviral therapy. BMC Pediatr.

[36] Yang C (2014). The impact of HAART initiation timing on HIV-TB co-infected patients, a retrospective cohort study. BMC Infect. Dis..

[37] Martinson NA, Moultrie H, van Niekerk R (2009). HAART and risk of tuberculosis in HIV-infected South African children: a multi-site retrospective cohort. Int. J. Tuberc. Lung Dis..

[38] Miranda A (2007). Impact of antiretroviral therapy on the incidence of tuberculosis: the Brazilian experience, 1995–2001. PLoS ONE.

